# Enhanced recovery after elective caesarean: a rapid review of clinical protocols, and an umbrella review of systematic reviews

**DOI:** 10.1186/s12884-017-1265-0

**Published:** 2017-03-20

**Authors:** Ellena Corso, Daniel Hind, Daniel Beever, Gordon Fuller, Matthew J. Wilson, Ian J. Wrench, Duncan Chambers

**Affiliations:** 1 School of Medicine and Dentistry, University of Sheffield, Sheffield, UK; 2 Clinical Trials Research Unit, Regent Court, 30 Regent St, Sheffield, S1 4DA UK; 3 Sheffield Teaching Hospitals Trust, Royal Hallamshire Hospital, Glossop Road, Sheffield, S10 2JF UK; 4 School of Health and Related Research, University of Sheffield, Regent Court, 30 Regent St, Sheffield, S1 4DA UK

**Keywords:** Enhanced recovery, Elective caesarean, Length of stay

## Abstract

**Background:**

The rate of elective Caesarean Section (CS) is rising in many countries. Many obstetric units in the UK have either introduced or are planning to introduce enhanced recovery (ER) as a means of reducing length of stay for planned CS. However, to date there has been very little evidence produced regarding the necessary components of ER for the obstetric population. We conducted a rapid review of the composition of published ER pathways for elective CS and undertook an umbrella review of systematic reviews evaluating ER components and pathways in any surgical setting.

**Methods:**

Pathways were identified using MEDLINE, EMBASE and the National Guideline Clearing House, appraised using the Appraisal of Guidelines for Research and Evaluation (AGREE II) tool and their components tabulated. Systematic reviews were identified using the Cochrane Library and Database of Abstracts of Reviews of Effects (DARE) and appraised using The Grading of Recommendations Assessment, Development and Evaluation (GRADE). Two reviewers aggregated summaries of findings for Length of Stay (LoS).

**Results:**

Five clinical protocols were identified, involving a total of 25 clinical components; 3/25 components were common to all five pathways (early oral intake, mobilization and removal of urinary catheter). AGREE II scores were generally low. Systematic reviews of single components found that minimally invasive Joel-Cohen surgical technique, early catheter removal and post-operative antibiotic prophylaxis reduced LoS after CS most significantly by around half to 1 and a half days. Ten meta-analyses of multi-component Enhanced Recovery after Surgery (ERAS) packages demonstrated reductions in LoS of between 1 and 4 days. The quality of evidence was mostly low or moderate.

**Conclusions:**

Further research is needed to develop, using formal methods, and evaluate pathways for enhanced recovery in elective CS. Appropriate quality improvement packages are needed to optimise their implementation.

**Electronic supplementary material:**

The online version of this article (doi:10.1186/s12884-017-1265-0) contains supplementary material, which is available to authorized users.

## Background

Caesarean Section (CS) is one of the most commonly performed operations worldwide. In many countries there is evidence that planned or “elective” operations account for a growing proportion of all CS [1–5]. In financial year 2012–13, 25.5% (167,283) of all deliveries in England were by CS, a rate that has almost doubled since 1993 [6]. Of these 75,621, or 45%, were elective operations.

The rate of elective CS continues to rise, despite initiatives to counter this trend. Birth by CS is associated with prolonged hospital stay in comparison to spontaneous birth and the majority of women remain in hospital for at least two days after a planned CS procedure [6]. The perioperative management of birth by planned CS and postoperative care therefore represents a substantial care and cost burden to the NHS. Crucially, the majority of women undergoing these elective procedures are young and fit. Not only do they possess the capacity for rapid recovery, but the birth of a new baby is an unique incentive to return quickly to “normal” function. Hospital discharge for this group of women the day after surgery could potentially halve in-patient stay; result in a substantial reduction in care burden and cost savings for obstetric units. Considering the current financial pressures on the NHS it is not surprising therefore, that there is widespread interest in the UK in introducing enhanced recovery (ER) for planned CS to facilitate earlier discharge [7].

The concept of an enhanced recovery programme following elective surgery is not new [8]. The aim of enhanced recovery is to optimise multiple aspects of patient care to improve recovery thereby facilitating earlier discharge, without a reduction in patient satisfaction or the quality of care [9–13]. Much of the work establishing the benefits of enhanced recovery concerned patients undergoing Colorectal Surgery, with other specialties, such as Gynaecology, Urology and Orthopaedics, adopting the concept later [13, 14]. This widespread adoption is no doubt related to the mounting evidence that the implementation of enhanced recovery programmes result in benefits such as reduced morbidity, reduced length of stay and an earlier return to normal activities for patients [9, 13]. Allowing patients to go home the day after an elective caesarean section is supported by recommendations from national guidance. In the UK, The National Institute for Health and Care Excellence states that, “women who are recovering well, are apyrexial and do not have complications following CS should be offered early discharge (after 24 h) from hospital and follow-up at home, because this is not associated with more infant or maternal readmissions.” [15] Components of enhanced recovery pathways include the provision of preadmission information to expectant mothers, good perioperative nutrition and hydration, minimally invasive surgical techniques, efforts to maintain normothermia, prevention of postoperative nausea, early removal of urinary catheters, effective postoperative pain relief that does not solely depend on systemic opioids, rapid postoperative mobilisation and a coordinated perioperative care pathway designed and managed by a multidisciplinary team [13, 16]. However there is great variation between pathways even within particular surgical populations [17].

Generally systematic reviews of therapeutic interventions evaluate whether, over several Randomised Controlled Trials (RCTs), a consistent treatment effect is observed for one intervention over another, and whether that treatment effect is worthwhile. This is useful when there is a well-developed and stable intervention that all stakeholders agree is a potentially useful approach to a clinical problem. Sometimes, the most suitable intervention to test has yet to be established; this is particularly the case in complex interventions, those with several interacting components [18]. There may be a variety of candidate intervention components, which people have already tried in different combinations [19–21]. When this is the case, systematic reviews can be used to inform the content of interventions [22] and identify appropriate outcomes for their future evaluation [23].

This paper presents two pieces of work intended to inform synthesis of a new clinical pathway for enhanced recover after elective CS. The first element is a rapid review of published clinical pathways for enhanced recovery after elective CS, recording which components are used in each. Rapid reviews involve explicit and rigorous method approaches but involve concessions compared to standard systematic review procedures, typically due to resource constraints [24, 25]. The second element is an overview or umbrella review of systematic reviews evaluating individual components or packages of components intended to enhance recovery after surgery of any kind. The purpose of the second component is to understand the effect on length of hospital stay of individual components and component packages, as well as to record what clinical outcomes are used by systematic reviews. This work has been undertaken in order to: (1) help patients and clinicians design an optimal enhanced recovery pathway to further inform policy-makers, clinicians and patients in obstetric units across the NHS; and, (2) inform the design of future research into the evaluation of enhanced recovery pathways for elective CS.

## Methods

### Protocol and registration

Members of the team developed the methods for this review. On 23rd October 2014, the review protocol was submitted for registration with the International Prospective Register of Systematic Reviews (PROSPERO) database, and was registered on 24 Oct 2014 with registration number CRD42014014458.

### Eligibility criteria

1. Enhanced Recovery after Surgery (ERAS) Packages in Elective Caesarean

Eligible studies included research articles or conference abstracts proposing or detailing components of a clinical pathway for enhanced recovery after elective caesarean section. There were no restrictions on study design and no date limits. Studies that were not published in English were excluded.

2. Systematic Reviews of ERAS components and packages in any setting

Eligible studies included Cochrane or other systematic reviews evaluating one or more component(s) aimed at reducing length of hospital stay following elective surgery. As the focus of the study is the identification of components for enhanced recovery after elective caesarean, systematic reviews that did not evaluate an ERAS package or component(s) were excluded. We did not include other umbrella reviews, but, when we identified them, did scan them for eligible systematic reviews.

### Literature search

1. ERAS Packages in Elective Caesarean

Ovid MEDLINE 1946 to October 19, 2014, and EMBASE 1974 to October 19, 2014, were searched for any paper detailing a clinical pathway for enhanced recovery after elective caesarean section. The MEDLINE search strategy is available in Additional file 1. The National Guideline Clearing house was also searched for guidelines relating to “enhanced recovery”.

2. Systematic Reviews of ERAS components and packages in any setting

The Cochrane Library was searched for Cochrane or other systematic reviews using the search terms “enhanced recovery” and “length of stay”, “umbilical cord clamping”, “early catheter removal”, “early fluids and food”, “caesarean section” and “surgical incision”, and finally “perioperative hypothermia”. The Database for Abstracts of Reviews of Effects (DARE) was also searched for systematic reviews relating to “enhanced recovery” from 2013 to 2014.

### Study selection

One researcher (EC or DB) independently screened citations at title and abstract according to the eligibility criteria above. Papers found to be eligible at title/abstract were retrieved and screened at full paper. Eligibility queries were resolved by a second researcher (DH / IW).

### Data collection process

One member of the review team (EC or DB) extracted the data, a second (DH) resolved queries.

### Data extraction

1. ERAS Packages in Elective Caesarean

For the proposed ERAS packages in elective caesarean, components recommended in each article were tabulated by phase of operation (pre-, intra- and post-operative).

2. Systematic Reviews of ERAS components and packages in any setting

For the overview of reviews, data on the composition of ERAS packages were not extracted; relatively up-to-date information on the components used in most of the trials included in ERAS reviews can be found in the umbrella review by Paton and colleagues [17, 26]. Primary and secondary outcomes reported by each eligible systematic review were tabulated. For each systematic review, summary of findings tables concerning length of stay data (measured in days, with a 95% confidence interval), were aggregated into a single table. Where the systematic review did not have a summary of findings table, the data was extracted to create one.

### Risk of bias in individual studies

1. ERAS Packages in Elective Caesarean

Eligible clinical pathways for enhanced recovery after elective caesarean were assessed using the Appraisal of Guidelines for Research & Evaluation (AGREE II) Instrument [27].

2. Systematic Reviews of ERAS components and packages in any setting

Eligible systematic reviews were assessed by a single reviewer (EC or DB) using A Measurement Tool to Assess Systematic Reviews (AMSTAR) [28], a reliable and valid 11-item tool. The assessments were checked and queries were resolved by a second reviewer (DH). Quality assessment was not performed for on-going systematic reviews as not all quality criteria can be appraised before publication.

### Risk of bias across studies

For the summary of findings table, data on length of stay (extracted from Cochrane systematic reviews) was assessed using The Grading of Recommendations Assessment, Development and Evaluation (GRADE) tool [29]. GRADE is widely used in Cochrane and other systematic reviews and was developed with the involvement of leading members of the Cochrane Collaboration. Where systematic reviews had been published without GRADE tables, EC or DB extracted Length of Stay data from the reviews and assessed quality of evidence using GRADE. DH checked their summaries.

### Summary measures

There were no summary measures for the review of clinical pathways for enhanced recovery in elective caesarean. For the overview of reviews, the length of stay, measured in days, with 95% confidence interval, were reported in the aggregated summary of findings table.

### Synthesis of results

A narrative summary of tabulated data was provided. No statistical synthesis was undertaken.

## Results

### Literature search

1. ERAS Packages in Elective Caesarean

The results of the literature search are presented in Additional file 2: Figure S1. This search strategy yielded 40 studies. After removing duplicates, 35 unique papers remained, eight of which were deemed ‘eligible at title/abstract’. Of these eight papers, three [30–32] met the inclusion criteria at full text. Of the remaining five, four were conference abstracts [33–36] covering two studies, which were not retrievable at full text but were included in the study as relevant qualitative data (reported enhanced recovery components in elective caesarean) were extracted from the abstract. The other was a poster, also included, which was provided via correspondence with the author of one of the studies.

2. Systematic Reviews of ERAS components and packages in any setting

The results of this literature search are presented in Additional file 3: Figure S2. A total of 126 papers were identified. After removing duplicates, 115 unique papers were retrieved from the search of the databases and through additional records (Cochrane *n =* 76, DARE *n =* 23, other *n =* 16), of which 51 were deemed ‘eligible at title/abstract’. Five of the Cochrane citations were excluded at full paper, as they did not evaluate a relevant ERAS component. 45 eligible studies remained (two papers covered the same study [37, 38], with one provided via correspondence with the author [38]) and were included for data extraction [12, 37–81]. 24 of the eligible studies reported quantitative analyses of Length of Stay (LoS) data (Cochrane *n =* 15, DARE *n =* 3, other *n =* 6). Although 39 studies included quantitative length of stay data, only 24 studies were included in LoS data extraction as the remaining 15 studies provided insufficient information on length of stay, for example ranges rather than standard deviations. Out of the additional 16 papers, 13 were identified for inclusion from the umbrella review by Paton et al. [17]. Ineligible papers and reasons are detailed in Additional file 4: Table S1.

### Data extraction

1. ERAS Packages in Elective Caesarean

The five studies reporting ERAS packages in elective caesarean were rapidly reviewed to determine the most common package components. The most common ERAS components were early oral intake (*n =* 5), early mobilization (*n =* 5), early removal of catheters (*n =* 5), patient advice and information (*n =* 4) and regular postoperative analgesia (*n =* 4). Components such as venous thromboembolism risk assessment, preoperative and intraoperative fluid balance, breastfeeding education, patient warming, delayed umbilical cord clamping, and prevention of postoperative nausea and vomiting were less common. The results are displayed in Additional file 5: Table S2.

2. Systematic Reviews of ERAS components and packages in any setting

For the overview of reviews, qualitative data on primary and secondary outcomes addressed by each eligible systematic review have been tabulated (Additional file 6: Table S3). The five most common outcomes were post-operative complications (*n =* 40), length of stay (*n =* 39), process outcomes (*n =* 37), survival (*n =* 28) and functional outcomes (*n =* 24). The five most reported outcomes (i.e. where data was available) were length of stay (*n =* 37), post-operative complications (*n =* 36), process outcomes (*n =* 30), survival (*n =* 22) and functional outcomes (*n =* 19). The review by Paton and colleagues has already noted that there is considerable variation in the definition of our outcome of interest, with only some reviews distinguishing between length of stay as primary hospital stay (the number of days in hospital after surgery) and total length of stay (total days spent in hospital including any readmissions) [17]. There were other discrepancies of terminology which are detailed in the table of outcomes (Additional file 6: Table S3).

Length of stay data (measured in days, with a 95% confidence interval) was extracted from the 24 eligible systematic reviews. The quality of evidence was assessed on a scale from “very low” to “high” quality evidence using the GRADE tool. Components demonstrating the greatest impact on length of stay in any surgical setting were delayed umbilical cord clamping (CS / vaginal birth – infant LoS (assumed to be relevant to the mother) reduced by 16.40 days – low quality evidence) [43], minimally invasive Joel-Cohen surgical technique (CS – LoS reduced by 1.5 days – very low quality evidence) [41], preoperative immune enhancing drinks (gastro-intestinal surgery – LoS reduced by 0.97 days - moderate quality evidence) [40] and early postoperative fluids / food (colorectal surgery – LoS reduced by 0.89 days – moderate quality evidence) [46]. Length of stay data was also extracted from eligible ERAS packages and assessed for quality. ERAS showed a reduction in length of hospital stay in elective surgery (LoS reduced by 1.14 days – low quality evidence) [57], in colorectal surgery (greatest reduction in LoS was that reduced by 3.75 days – moderate quality evidence) [72], and in upper gastrointestinal surgery (LoS reduced by 2.28 days – moderate quality evidence) [59].

### Risk of bias in individual studies

1. ERAS Packages in Elective Caesarean

Two of the five eligible studies proposing ERAS packages for elective caesarean were rated moderate quality guidelines (Wrench [32] and Halder [31]) scoring 61% and 62% across the AGREE II domains respectively. The other eligible studies (Lucas [30], Damluji [33] and Abell / Long [34–36]) were rated low quality evidence as they only scored 39%, 28% and 30% respectively across the domains. The full details of quality assessment are tabulated in Additional file 7: Table S4.

2. Systematic Reviews of ERAS components and packages in any setting

Overall the Cochrane systematic reviews were of very high quality when rated according to the AMSTAR items. Systematic reviews that were retrieved from the DARE search were of lower quality but still scored as moderate quality evidence. The full details of quality assessment are tabulated in the appendix (Additional file 8: Table S5 and Additional file 9: Table S6).

### Risk of bias across studies

For the aggregated summary of findings table of length of stay data, studies were assessed for quality using the GRADE tool. Evidence ranged from very low quality (*n =* 7) to moderate quality (*n =* 8; low quality *n =* 9). Over a third of the studies indicated substantial heterogeneity and no blinding. Five of the systematic reviews only included a single study for length of stay data.

## Discussion

### Summary of findings

The rapid review identified five clinical protocols for ER after CS (all from the UK) involving a total of 25 clinical components [30–33]. Only 3/25 components were common to all five pathways (early oral intake, mobilization and removal of catheter) demonstrating considerable differences in the composition of enhanced recovery packages between UK maternity units. Of the systematic reviews evaluating single component ERAS interventions in CS, only minimally invasive Joel-Cohen surgical technique [41], early removal of catheter [47] and post-operative antibiotic prophylaxis [44] appeared to show effects that were statistically significant at the 5 percent level. These interventions reduced length of stay by 0.49 to 1.5 days (catheter removal was presented as increased length of stay for indwelling catheter [47]), but the quality of evidence was moderate at best.

Since only a minority of ER interventions for CS have been subject to systematic review, reviews of ER interventions / packages from other settings were included. The rationale for their inclusion was to allow expert decision-makers to assess the functional logic and assumed mechanisms of action associated with particular interventions and determine whether they should be deemed transferable from one surgical setting to another. For example, whilst ER for colorectal cancer targets optimising compromised perioperative bowel function in an older population function, ER interventions for elective CS are deployed in a younger population without compromised digestive tract.

Of those reviews evaluating components in any other surgical setting, almost all (except patient warming [42] and delayed umbilical cord clamping [43], of which the latter, for infants only, actually showed the most significant LoS reduction) demonstrated statistically significant effects at the 5 percent level. These interventions reduced length of stay by 0.3 to 0.97 days, but again, the quality of evidence was moderate at best. Meta-analysis was undertaken in ten systematic reviews of multi-component ERAS packages (eight for colorectal surgery, one for upper gastrointestinal surgery and one for all elective surgery), which consistently demonstrated results that were statistically significant at the 5 percent level. Mean effect sizes for LoS were larger in reviews of ERAS packages than in reviews of single components (median reduction in length of stay versus control of −2.45 days and median range −3.28 days to −1.45 days, values rounded to two decimal places), indicating the likelihood that multiple components provide additive effects. The quality of evidence was generally low.

### Strengths and limitations of review

Our study used multiple approaches to identify and synthesise evidence relevant to the design of enhanced recovery programmes for elective CS. Systematic review of complex interventions is challenging [82], and there are areas where a better resourced study might have better described the complexity of the primary data. Where systematic reviews investigated the effect of individual components, it was rarely clear what other clinical components that might affect length of stay might be present in the included trials.

ERAS packages evaluated in systematic reviews varied greatly in the number of individual components employed. For instance 23 RCTs in one systematic review [57], used a minimum of four and a maximum of 13 out of 21 components. Most of the systematic reviews of pathways did not make it clear whether there was flexibility over the delivery of research and control interventions in the included trials and, if so, how much leeway was available to professionals in adapting pathways to local circumstances [83, 84]. Similarly, Paton and colleagues make the point that components of elements of early enhanced recovery pathways, become widely adopted in usual care, making the synthesis of studies from different time points questionable [17].

Rapid reviews generally employ methods that are rigorous and transparent, with compromises on best practice made because of time and resource constraints [24, 25, 85, 86]. There is little guidance on which methodological concessions should be sanctioned, although the use of more limited search strategies is one generally thought legitimate [25]. We searched the Cochrane Library and DARE for systematic reviews to maximise the completeness and timeliness of the search without being burdened with an excessively large number of references for screening. In this, and other respects, our umbrella review of systematic reviews broadly followed the methods recommended by the Cochrane Collaboration for overviews of systematic reviews and in some cases went beyond them [87]. The process followed a protocol specified in advance and published via PROSPERO [88]. As recommended, we used a validated tool, AMSTAR [28], widely used in previous overviews, to assess the limitations of included systematic reviews. An assessment of the overall quality of evidence is another important feature of systematic review overviews. For Cochrane overviews it is recommended to assess the quality of evidence across reviews based on GRADE assessments in the included systematic reviews; where these were not present, we performed our own GRADE assessments of the quality of evidence for length of stay [29].

Data collection was limited to our primary outcome of interest; we did not seek to obtain additional data, for example by contacting authors, because of resource constraints. Similarly, although differences in the reporting of length of stay were noted, we were not able to investigate these in any depth. A further potential limitation was our restriction of the searches to two indexed databases and only one source of grey literature to identify other enhanced recovery pathways for elective CS in grey literature. English language restrictions also meant that we knowingly, excluded one relevant article [89]. The exclusion of non-English language papers from systematic reviews is not thought to bias estimates of effectiveness for most types of intervention [90]. The abstract of the single non-English language clinical pathway publication which we noted, seemed to confirm the findings of other studies, that enhanced recovery pathways reduce length of hospital stay after elective CS, without safety concerns or increased readmissions [89].

The searches for our umbrella review were run 18 months after those of the last umbrella review of the enhanced recovery after surgery [17, 26], work that was better resourced than our own. Our review identified an additional 28 systematic reviews on ERAS to the seventeen included by Paton and colleagues [17]. Unlike the Paton review, we also included reviews evaluating single intervention components, rather than entire pathways. Shortcomings of our umbrella review are that we did not investigate overlap between eligible systematic reviews or search for relevant RCTs not included by the reviews. Whether reviews are contemporary (‘up-to-date’) is often neglected in overviews [91], but the risk of bias to our own is small, with the Paton review of RCTs itself so recently conducted [26]. However, overlap of included studies between systematic reviews of ERAS packages has been shown to be substantial for colorectal surgery [26].

Differences in resourcing aside, the methods used in our review and the Paton review reflect the differences in objectives, with theirs undertaken to inform commissioning of services and further research, and ours to inform intervention synthesis [22]. In other words, our aim was to inform the development of an enhanced recovery pathway for elective CS by reference to the best available evidence for enhanced recovery components and pathways in elective CS and other settings. Overall, the sample of pathways we reviewed can be considered fit-for-purpose in that it shows a diversity of intervention content which is nonetheless limited in scope when compared with ERAS programmes from other clinical settings, signalling the need for consensus work.

### Implications for health professionals, policymakers and patients

Given that enhanced recovery programmes were developed and implemented initially for patients undergoing cancer surgery, not all the interventions we review in the umbrella review are relevant to enhanced recovery in elective CS. However, by adopting a broad scope, we have ensured we offer decision-makers for a consensus exercise (see below) information on the widest range of potentially relevant interventions, to inform the production of durable guidelines [92]. Additional file 10: Figure S3 and Additional file 11: Table S7 presents a programme theory of the proposed mechanisms of action for broad categories of ERAS components, and how they might work together in an enhanced recovery pathway.

The adoption of recommended healthcare innovations is neither a linear process nor guaranteed by the availability of robust evidence, with many other factors influencing the change process [93]. The Paton review dedicated a chapter to studies charting the implementation of ER pathways [17], identifying facilitators which are summarised in Additional file 12: Table S8, mapped to a classes of knowledge transfer targets from the taxonomy by Wensing and colleagues [94]. These themes are echoed by recent [95–97] and ongoing [98] theory-based quality improvement studies as important considerations in the reliable implementation of a clinical pathway. Of particular interest is the tension between the desire to implement clinical protocols rigidly, and the requirement by some stakeholders or centres, to maintain a flexibility of approach based on the needs of particular patients or due to local circumstances [99, 100]. These tensions have wider resonances in the differences between those who advocate the demonstration of fidelity in the implementation of complex interventions [101–103] and those who favour adaptation of complex interventions to circumstance [84, 104–106]. Those designing both clinical pathways and further research need to consider carefully which clinical and quality improvement components are essential and on which flexibility of approach can be permitted.

### Further research

A meeting of women who have given birth by elective-CS, midwives, obstetricians, obstetric anaesthetists, neonatologists and Quality Improvement (QI) experts, held in London on 5th March 2015, generated a consensus on a package of core clinical and QI components for enhanced recovery. The resulting guideline, which will be current, specific, clinically relevant, based on an up-to-date evidence base and patient-important outcomes [107], will be reported separately. There is currently a paucity of existing evidence to support structured ER interventions in CS, the components of such interventions are heterogeneous and their effect has not yet been investigated in the setting of controlled studies. However, it is reasonable to say that there is wide acceptance that ER pathways in general are useful quality improvement tools to streamline surgical care, despite variation in pathways between institutions resulting from incorporation of local expertise. The challenge for future research will be the rigorous study of the facilitators and barriers to the implementation of ER in CS, monitoring the adoption and spread of the principles of ER as they embed into practice and the search for evidence of a permanent improvement in quality of care over time. Further work should concentrate on these domains to meaningfully assess the impact of the intervention and establish if its impact is sustained.

## Conclusions

The composition of clinical pathways for enhanced recovery after elective CS varies radically and their design is based neither on current best evidence nor best practice for guideline development. Systematic reviews provide varying quality evidence that some individual components can reduce length of stay by about, most commonly, half a day after CS. Although this represents a substantial proportional reduction in duration of hospital stay after CS, packages of components for enhanced recovery in other settings can achieve much greater absolute reductions in length of stay of between one and 4 days. Further research is needed to develop and evaluate pathways for enhanced recovery in elective CS with appropriate quality improvement packages to optimise their implementation.

## Additional files

Additional file 1: MEDLINE Search Strategy. This file provides a reproduction of the search strategy used in the MEDLINE search. (PDF 12 kb)
Additional file 2: Figure S1. Eligible studies for ERAS packages in elective caesarean. Flow diagram of the study selection process for ERAS packages in elective caesarean. (PDF 33 kb)
Additional file 3: Figure S2. Eligible systematic reviews for ERAS components and packages in any setting. Flow diagram of the study selection process for ERAS components and packages in any setting. (PDF 36 kb)
Additional file 4: Table S1. Excluded studies. Table listing the studies found to be ineligible at full reading (ERAS components and packages in any setting). (PDF 10 kb)
Additional file 5: Table S2. Components of pathways for enhanced recovery after elective CS. Table listing the various enhanced recovery components found in the included studies. (PDF 22 kb)
Additional file 6: Table S3. Outcomes reported by systematic reviews of components and pathways for ERAS. Table listing the various outcomes from the included studies, presented by category and in specific detail. (PDF 57 kb)
Additional file 7: Table S4. AGREE II criteria for assessing quality of studies of proposed ERAS packages in elective caesarean. Table presenting the results of the quality assessment undertaken in terms of risk of bias of the included studies (ERAS packages in elective caesarean). (PDF 22 kb)
Additional file 8: Table 5. Aggregate Summary of Findings table. Table presenting the summary of findings from the included studies (ERAS components and packages in any setting). (PDF 57 kb)
Additional file 9: Table 6. AMSTAR Assessment. Table presenting the results of the quality assessment undertaken in terms of risk of bias of the included studies (ERAS components and packages in any setting). (PDF 45 kb)
Additional file 10: Figure S3. Proposed mechanisms of action for broad categories of ERAS components. Figure illustrating how the proposed mechanisms of action may work in an enhanced recovery pathway. (PDF 212 kb)
Additional file 11: Table S7. Interventions, mechanisms and actions. Table detailing the theory of the proposed mechanisms of action in an enhanced recovery pathway. (PDF 22 kb)
Additional file 12: Table S8. Proposed barriers and facilitators of uptake. Table detailing the barriers and facilitators of adoption of healthcare innovations. (PDF 19 kb)

## Abbreviations

AGREE II: Appraisal of guidelines for research and evaluation
AMSTAR: A measurement tool to assess systematic reviews
CS: Caesarean section
DARE: Database of abstracts of reviews of effects
ER: Enhanced recovery
ERAS: Enhanced recovery after surgery
GRADE: The grading of recommendations assessment, development and evaluation
LoS: Length of stay
PROSPERO: International prospective register of systematic reviews
QI: Quality improvement
RCTs: Randomised controlled trials
