# Supplementary material for: Enhanced recovery after elective caesarean: a rapid review of clinical protocols, and an umbrella review of systematic reviews
Source: BMC Pregnancy Childbirth. 2017 Mar 20;17:91. doi: 10.1186/s12884-017-1265-0 (PMC5359888; doi:10.1186/s12884-017-1265-0)
Supplement: Additional file 1: — MEDLINE Search Strategy. This file provides a reproduction of the search strategy used in the MEDLINE search. (PDF 12 kb) [file 12884_2017_1265_MOESM1_ESM.pdf]

Additional file 1 – MEDLINE Search Strategy

1. Cesarean Section/
2. caesarian section.mp.
3. cesarian section.mp.
4. cesarean section.mp.
5. caesarean section.mp.
6. or/1-5
7. limit 6 to (english language and humans)
8. pathway$.mp.
9. guideline$.mp.
10. recommendation$.mp.
11. protocol$.mp.
12. Practice guidelines/
13. critical pathway/
14. Critical Pathways/
15. Critical Pathways/ or care pathway.mp.
16. Critical Pathways/ or Integrated care pathway.mp.
17. Critical Pathways/ or care map.mp.
18. multidisciplinary approach.mp.
19. ((clinical or critical or care) adj path$).tw.
20. (care adj (map$ or plan$)).tw.
21. exp Guideline/
22. guideline.pt.
23. or/8-22
24. limit 23 to (english language and humans)
25. "enhanced recovery".mp.
26. "fast-track surg$".mp.
27. "perioperative care".mp.
28. Intraoperative Care/mt
29. Preoperative Care/mt
30. Perioperative Care/mt
31. Postoperative Care/mt
32. ERAS.tw.
33. (multimodal adj care).tw.
34. (multi-modal adj care).tw.
35. ((perioperative adj care) and enhanced).tw.
36. ((postoperative adj care) and accelerat$).tw.
37. or/25-36
38. limit 37 to (english language and humans)
39. 7 and 24 and 38
