# Supplementary material for: Enhanced recovery after elective caesarean: a rapid review of clinical protocols, and an umbrella review of systematic reviews
Source: BMC Pregnancy Childbirth. 2017 Mar 20;17:91. doi: 10.1186/s12884-017-1265-0 (PMC5359888; doi:10.1186/s12884-017-1265-0)
Supplement: Additional file 2: Figure S1. — Eligible studies for ERAS packages in elective caesarean. Flow diagram of the study selection process for ERAS packages in elective caesarean. (PDF 33 kb) [file 12884_2017_1265_MOESM2_ESM.pdf]

Additional file 2 – Fig 1: Eligible studies for ERAS packages in elective caesarean

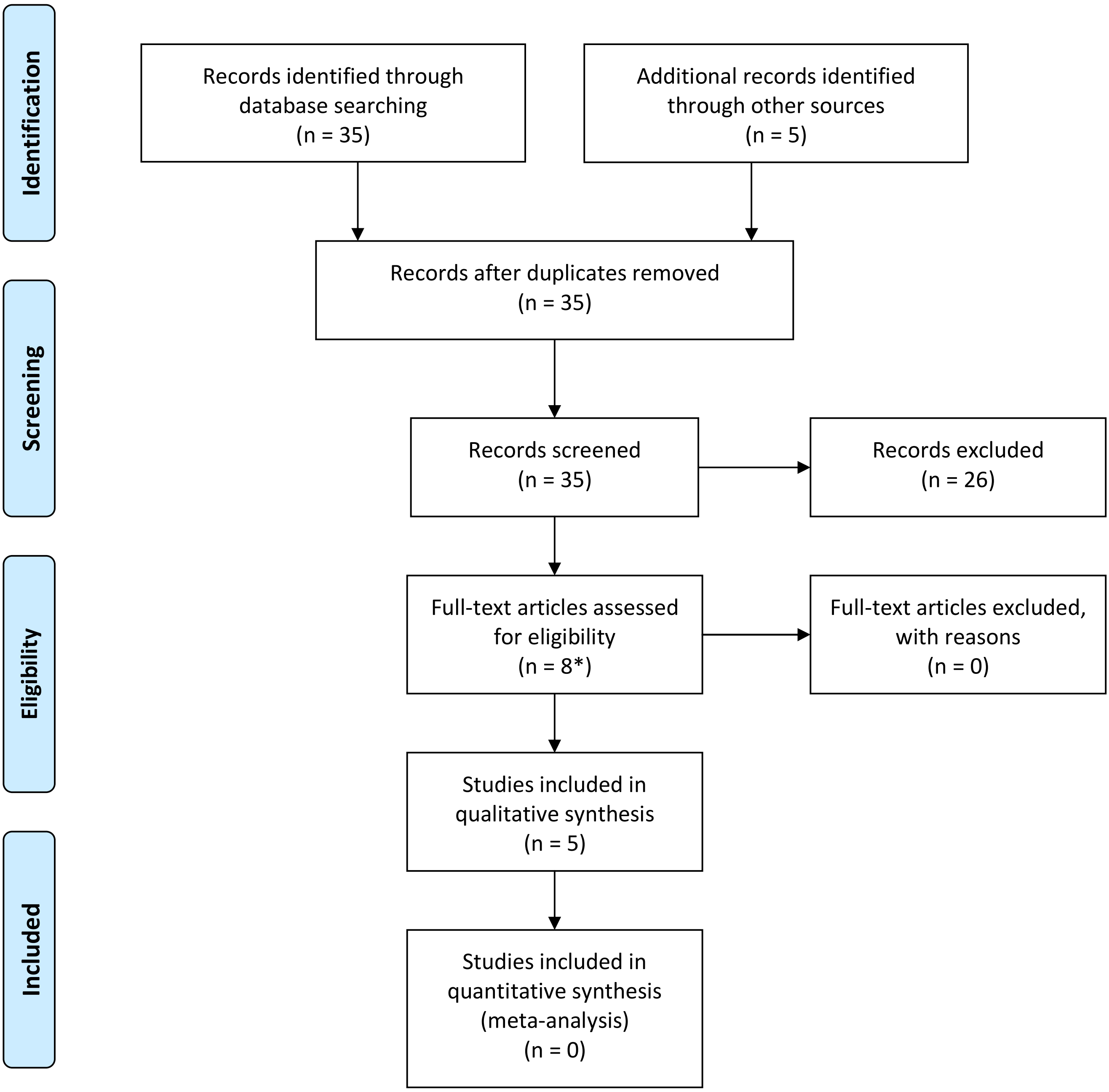

* Note that of the 8 full text articles, 5 covered 2 of the included studies, with the other 3 covering the other 3 studies – hence why only 5 studies are documented as being included in the qualitative synthesis.
