# Supplementary material for: Enhanced recovery after elective caesarean: a rapid review of clinical protocols, and an umbrella review of systematic reviews
Source: BMC Pregnancy Childbirth. 2017 Mar 20;17:91. doi: 10.1186/s12884-017-1265-0 (PMC5359888; doi:10.1186/s12884-017-1265-0)
Supplement: Additional file 3: Figure S2. — Eligible systematic reviews for ERAS components and packages in any setting. Flow diagram of the study selection process for ERAS components and packages in any setting. (PDF 36 kb) [file 12884_2017_1265_MOESM3_ESM.pdf]

Additional file 3 – Fig 2: Eligible systematic reviews for ERAS components and packages in any setting

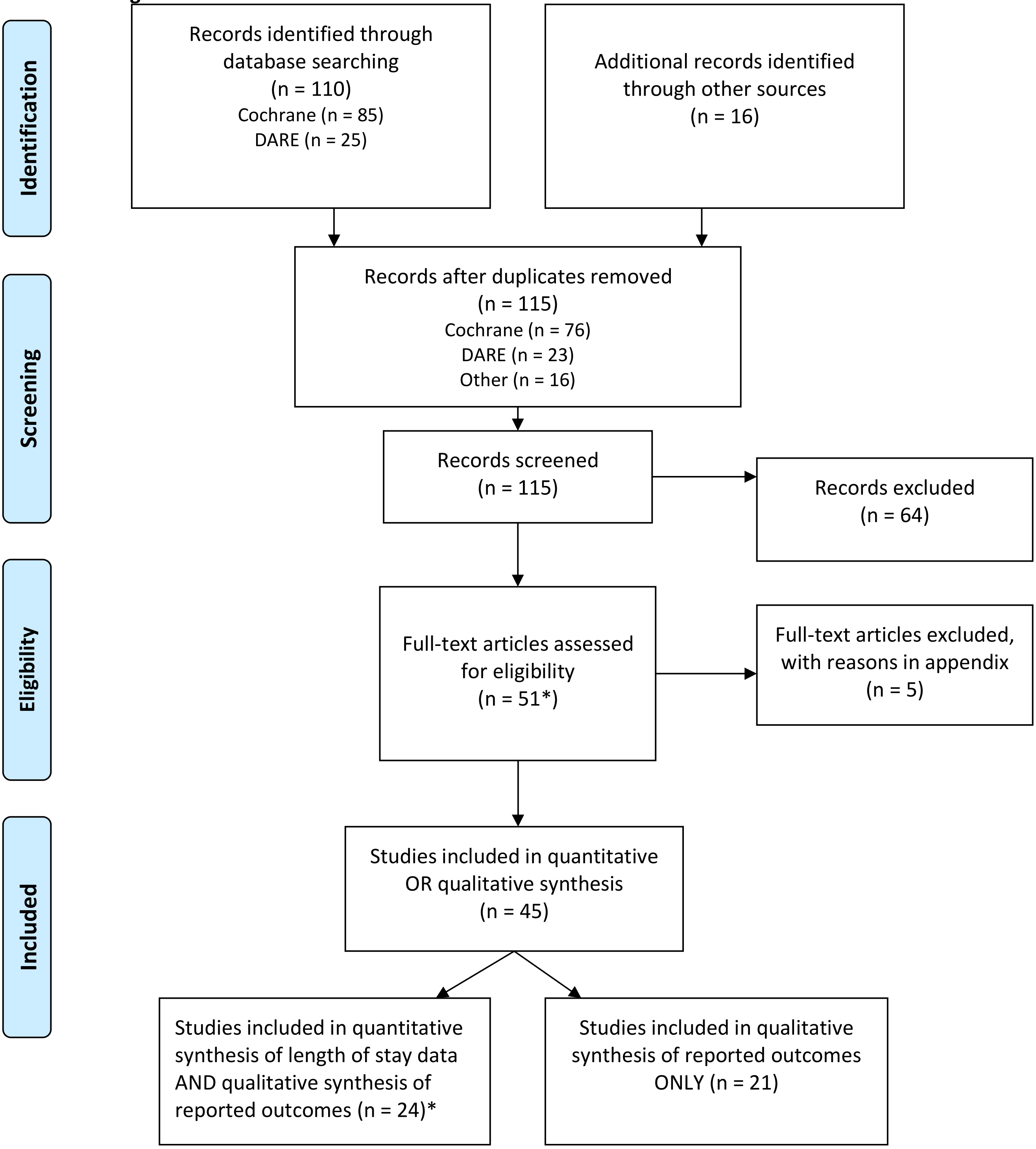

* Note that of the 51 full text articles, 2 covered 1 of the included studies, with the other 49 covering the other 49 studies – hence why only 45 studies are documented as being included in the quantitative or qualitative synthesis.
