# Supplementary material for: Enhanced recovery after elective caesarean: a rapid review of clinical protocols, and an umbrella review of systematic reviews
Source: BMC Pregnancy Childbirth. 2017 Mar 20;17:91. doi: 10.1186/s12884-017-1265-0 (PMC5359888; doi:10.1186/s12884-017-1265-0)
Supplement: Additional file 4: Table S1. — Excluded studies. Table listing the studies found to be ineligible at full reading (ERAS components and packages in any setting). (PDF 10 kb) [file 12884_2017_1265_MOESM4_ESM.pdf]

Additional file 4 – Table 1: Excluded studies

Author name	Reason for exclusion
Vasudevan [108]	Not an evaluation of a relevant ERAS component.
Burry [109]	
Chatmongkolchart [110]	
McDonald [111]	
Soltani [112]	

References

108. Vasudevan C, McGuire W. Early removal versus expectant management of central venous catheters in neonates with bloodstream infection. Cochrane database Syst Rev. 2011;(8):CD008436.

109. Burry L, Rose L, McCullagh IJ, Fergusson DA, Ferguson ND, Mehta S. Daily sedation interruption versus no daily sedation interruption for critically ill adult patients requiring invasive mechanical ventilation. Cochrane database Syst Rev. 2014;(7):CD009176.

110. Chatmongkolchart S, Prathep S. Supplemental oxygen for caesarean section during regional anaesthesia. Cochrane database Syst Rev. 2013;(6):CD006161.

111. McDonald S, Abbott JM, Higgins SP. Prophylactic ergometrine-oxytocin versus oxytocin for the third stage of labour. Cochrane database Syst Rev. 2004;(1):CD000201.

112. Soltani H, Poulose TA, Hutchon DR. Placental cord drainage after vaginal delivery as part of the management of the third stage of labour. Cochrane database Syst Rev. 2011;(9):CD004665.
