# Supplementary material for: Enhanced recovery after elective caesarean: a rapid review of clinical protocols, and an umbrella review of systematic reviews
Source: BMC Pregnancy Childbirth. 2017 Mar 20;17:91. doi: 10.1186/s12884-017-1265-0 (PMC5359888; doi:10.1186/s12884-017-1265-0)
Supplement: Additional file 5: Table S2. — Components of pathways for enhanced recovery after elective CS. Table listing the various enhanced recovery components found in the included studies. (PDF 22 kb) [file 12884_2017_1265_MOESM5_ESM.pdf]

**Additional file 5 – Table 2: Components of pathways for enhanced recovery after elective CS**

| Phase of operation | Enhanced Recovery Components | **Lucas** [30] | **Wrench** [32] | **Halder** [31] | **Damluji** [33] | **Abell / Long** [34–36] | **Frequency** |
|---|---|---|---|---|---|---|---|
| Pre | Patient selection | - | ✓ | - | ✓ | - | 2 |
| | Patient advice and information | ✓ | ✓ | ✓ | - | ✓ | 4 |
| | VTE risk assessment | - | - | ✓ | - | - | 1 |
| | Clear fluids | - | ✓ | ✓ | - | - | 2 |
| | Carbohydrate drink | - | ✓ | ✓ | ✓ | - | 3 |
| | Fluid balance | - | ✓ | - | - | - | 1 |
| | Hemoglobin optimization | ✓ | - | ✓ | - | - | 2 |
| | Initiate breastfeeding teaching | ✓ | - | - | - | - | 1 |
| | Reduced fasting times | - | - | - | - | ✓ | 1 |
| Intra | Fluid balance | - | ✓ | - | - | - | 1 |
| | Prophylactic antibiotics | ✓ | - | ✓ | - | - | 2 |
| | Venous thromboprophylaxis | ✓ | - | ✓ | - | - | 2 |
| | Minimally invasive surgical technique | - | ✓ | ✓ | - | - | 2 |
| | Patient warming | - | ✓ | - | - | - | 1 |
| | Delayed cord clamping | - | ✓ | - | - | - | 1 |
| | Analgesia | ✓ | ✓ | ✓ | - | ✓ | 4 |
| Post | Early oral intake | ✓ | ✓ | ✓ | ✓ | ✓ | 5 |
| | Early mobilization | ✓ | ✓ | ✓ | ✓ | ✓ | 5 |
| | Early removal of catheter | ✓ | ✓ | ✓ | ✓ | ✓ | 5 |
| | Regular analgesia | ✓ | ✓ | ✓ | - | - | 3 |
| | Prevention of post operative nausea and vomiting | - | ✓ | - | - | - | 1 |
| | Debriefing of patient | - | - | ✓ | ✓ | - | 2 |
| | Early skin to skin contact (support to establish breastfeeding) | ✓ | ✓ | ✓ | - | - | 3 |
| | Community support (midwife visits, physiotherapists etc) | ✓ | - | ✓ | - | ✓ | 3 |
| | Opportunity to go home on day one | - | - | - | - | ✓ | 1 |
