# Supplementary material for: Enhanced recovery after elective caesarean: a rapid review of clinical protocols, and an umbrella review of systematic reviews
Source: BMC Pregnancy Childbirth. 2017 Mar 20;17:91. doi: 10.1186/s12884-017-1265-0 (PMC5359888; doi:10.1186/s12884-017-1265-0)
Supplement: Additional file 6: Table S3. — Outcomes reported by systematic reviews of components and pathways for ERAS. Table listing the various outcomes from the included studies, presented by category and in specific detail. (PDF 57 kb) [file 12884_2017_1265_MOESM6_ESM.pdf]

Additional file 6 – Table 3: Outcomes addressed by systematic reviews of components and pathways for ERAS

	Length of Stay	Survival	Functional outcomes	Compliance	Surgical outcomes	Anaesthetic outcomes	Post-operative complications	Post-operative pain	Process outcomes	Breastfeeding	Fetal & childhood wellbeing	Other
**Smith: Carbohydrate drink (elective surgery)** [39]	✓	-	✓	-	-	-	✓	-	-	-	-	✓
**Burden: Nutrition support (GI surgery)** [40]	✓	✓	✓	-	-	-	✓	-	✓	-	-	-
**Mathai: Types of surgical incisions (caesarean section)** [41]	✓	-	✓b	-	✓	-	✓	✓b	✓b	✓	✓	✓b
**Alderson: Preventing hypothermia (elective surgery)** [42]	✓	✓b	✓	-	✓	-	✓	-	✓	-	-	-
**Rabe: Umbilical cord clamping (caesarean section / vaginal birth)** [43] a	✓d	✓b	✓b	-	-	-	✓b	-	✓b	-	✓	✓b
**Smaill: Antibiotic prophylaxis (caesarean section)** [44]	✓	-	-	-	-	-	✓	-	-	-	✓b	✓b
**Mangesi: Early oral fluids (caesarean section)** [45]	✓	-	✓	-	-	-	✓	✓	✓b	✓b	-	-
**Andersen: Early oral intake (colorectal surgery)** [46]	✓	✓	-	-	-	-	✓	-	-	-	-	-
**Abdel-Aleem: Early catheter removal (caesarean section)** [47]	✓	-	✓	-	✓b	-	✓	✓	✓b	-	-	-
**Billson: Nutrition intervention (ovarian cancer)** [48]	✓	✓	✓	-	-	-	✓	-	✓	-	-	-
**Dodd: Surgical techniques (caesarean section)** [49]	✓	✓	-	-	✓	-	✓	✓	✓	✓b	✓b	-
**Mackeen: Surgical technique (caesarean section)** [50]	✓	-	-	-	✓	-	✓	✓	✓	-	-	✓b
**McDonald: Umbilical cord clamping (caesarean section / vaginal birth)** [51] a	-	✓b	-	-	✓	-	✓	-	-	✓	✓	✓
**Urrútia: Preventing hypothermia (elective surgery)** [52] c	-	✓b	-	-	-	-	✓b	-	✓b	-	-	-
**Bamigboye: Closure vs. non closure of peritoneum (caesarean section)** [53]	✓	✓	✓	-	✓	-	✓	✓	✓	-	-	✓b
**Afolabi: Regional vs. general anaesthesia (caesarean section)** [54]	-	✓b	-	-	✓	-	✓	✓	✓	-	✓	-

	Length of Stay	Survival	Functional outcomes	Compliance	Surgical outcomes	Anaesthetic outcomes	Post-operative complications	Post-operative pain	Process outcomes	Breastfeeding	Fetal & childhood wellbeing	Other
**Charoenkwan: Early oral intake (gynaecologic surgery)** [55]	✓	-	✓	-	-	-	✓	-	✓	-	-	-
**Munday: Warming interventions (caesarean section)** [37, 38]	✓	-	✓	-	-	-	-	-	-	-	✓	-
**Ho: Sham feeding (colorectal surgery)** [56]	✓	-	✓	-	-	-	✓	-	✓	-	-	-
**Palethorpe: Alternative positions before umbilical cord clamping (caesarean section / vaginal birth)** [81] a, c	-	-	✓b	-	✓b	-	✓b	-	✓b	✓b	✓b	✓b
**Sia: Spinal anaesthesia (caesarean section)** [78]	-	-	-	-	-	✓	-	-	-	-	-	-
**Jacobs-Jokhan: Repair of uterine incision (caesarean section)** [79]	✓	-	✓b	-	✓	✓	✓	✓b	✓	✓b	-	✓b
**Becker: Early additional food and fluids (full-term infants)** [80]	✓b	-	-	-	-	-	-	-	-	✓	✓	-
**Nicholson (Elective surgery - pathway)** [57]	✓	✓	-	-	-	-	✓	-	✓	-	-	-
**Kagedan (Pancreatic surgery - pathway)** [58]	✓	✓	-	-	-	-	✓	-	✓	-	-	✓
**Markar (esophagectomy - pathway)** [59]	✓	✓	-	-	-	-	✓	-	✓	-	-	-
**Hughes (liver surgery - pathway)** [60]	✓	-	✓	✓	-	-	✓	-	-	-	-	-
**Coolsen (pancreatic surgery - pathway)** [61]	✓	✓	✓	✓	-	-	✓	-	✓	-	-	✓
**Zhuang (colorectal surgery - pathway)** [62]	✓	✓	✓	-	-	-	✓	-	✓	-	-	✓
**Spanjersberg (colorectal surgery - pathway)** [63]	✓	✓	✓	✓b	✓	-	✓	✓	✓	-	-	✓b
**Eskicioglu (colorectal surgery – pathway)** [64]	✓	✓	-	-	-	-	✓	-	✓	-	-	-
**Wind (colonic surgery – pathway)** [65]	✓	✓	✓	-	-	-	✓	✓	✓	-	-	-
**Adamina (colorectal surgery – pathway)** [66]	✓	-	-	-	-	-	✓	-	✓	-	-	-
**Ahmed (colorectal surgery – pathway)** [67]	✓	-	-	✓	-	-	-	-	✓	-	-	-
**Coolsen (liver surgery – pathway)** [68]	✓	✓	✓	✓	-	-	✓	-	✓	-	-	-

	Length of Stay	Survival	Functional outcomes	Compliance	Surgical outcomes	Anaesthetic outcomes	Post-operative complications	Post-operative pain	Process outcomes	Breastfeeding	Fetal & childhood wellbeing	Other
**Hall (hepatobiliary & pancreatic surgery – pathway)** [69]	✓	✓	✓	-	✓	-	✓	-	✓	-	-	-
**Lemmens (digestive surgery – pathway)** [70]	✓	✓	-	-	-	-	✓	-	✓	-	-	-
**Rawlinson (colorectal surgery – pathway)** [71]	✓	✓	-	-	-	-	✓	-	✓	-	-	-
**Varadhan (colorectal surgery – pathway)** [12]	✓	✓	-	-	-	-	✓	-	✓	-	-	-
**Walter (colorectal resections – pathway)** [72]	✓	✓	-	-	-	-	✓	-	✓	-	-	-
**Lv (colorectal surgery – pathway)** [73]	✓	✓	-	-	-	-	✓	-	✓	-	-	-
**Gouvas (colorectal surgery – pathway)** [74]	✓	✓	✓	-	-	-	✓	✓	✓	-	-	-
**Lv (gynaecological cancer – pathway)** [75] c	✓b	✓b	✓b	-	-	-	✓b	-	✓b	-	-	-
**Sturm (fast-track surgery – pathway)** [76]	✓	✓	✓	-	-	-	✓	✓	✓	-	-	-
**Khan (colorectal surgery & enhanced recovery – pathway)** [77]	-	-	-	-	-	-	-	-	✓	-	-	-
**Frequency**	39	28	24	5	12	2	40	12	37	7	9	13

a – Included additional outcomes that were not relevant to this review and were therefore excluded from the above table. **b** – Outcome listed in review but not reported; omission may relate to included studies not reporting on this outcome and is not necessarily a sign of poor review conduct. For outcome categories (e.g. "Post-operative complications") which cover a number of discrete outcomes, we have not flagged studies in this way if they have reported at least one such outcome, regardless that others may be omitted. **c** – No studies were included in the review / review was only at protocol stage. **d** – Length of stay provided for infant only (assumed to be relevant to mother).

Breakdown of Categories

Survival includes overall survival; mortality (b [42, 43, 51, 52, 54, 75]).

Functional outcomes include functional measures (b [48]); time to normal function; time to functional recovery; feeding; general well-being; fatigue (b [45]); return of gastrointestinal function (b [41, 75, 79]); time of first voiding; time to start of regular diet; mobilisation (b [41, 45, 79]); time out of bed; pulmonary function; time to first food; time to first oral fluid (b [45]); time to oral intake (b [41, 79]); nutrition aspects; emotional wellbeing (b [81]); immune system function; temperature; prevention of shivering; thermal comfort; psychological wellbeing (b [43]); infant bonding (b [43]); anxieties (b [43]).

Compliance includes compliance (b [63]); adherence.

Surgical outcomes include duration of surgery (b [47]); blood loss (b [81]); abdominal drains; intra-operative pain; intra-operative bladder injury (b [47]); operative procedure on wound; operative complications (b [41]).

Anaesthetic outcomes include inadequate pain control requiring general / supplemental anaesthesia; requirement for and amount of ephedrine used; nausea & / or vomiting; headache within seven days from spinal anaesthesia; time to dermatomal block (thoracic T4 level); occurrence of high dermatomal sensory block (above C8 level).

Post-operative complications include post-operative complications (b [75]); serious infectious complication; complication rate; adverse effects (b [42, 51, 52]); re-operation rate; post-operative infection (b [41, 54, 79]); need for re-laparotomy; re-catheterisation; wound infection (b [42, 52, 54]); wound complications (b [42, 52]); endometritis (b [54]); urinary tract infection (b [54, 55]); cardiovascular complications (b [52]); pulmonary complications; febrile morbidity; post-operative pyrexia (b [41]); anastomotic leakage; temperature; intraabdominal abscess; pneumonia; post-partum haemorrhage (b [43, 81]); post-operative morbidity (b [51]); post-operative sepsis; vomiting; nausea; blood transfusion (b [52, 81]); haemoglobin level & / or haematocrit level (b [41]); haematocrit level; anaemia (b [41, 79]); wound complications; deep vein thrombosis & / or pulmonary embolism; need for iron therapy in postnatal period (b [81]); pressure ulcers (b [42, 52]); burns (b [52]); bleeding complications (b [42]); abdominal pain (b [45]); acute myocardial infarction; thrombosis; bloating (b [45]); abdominal distention; ketosis (b [45]); paralytic ileus (b [53]); postoperative ileus; hypoglycaemia (b [45]); urinary bladder distention; retention of urine; haematoma; seroma; skin separation; hypertrophic scar; thromboembolic complications (b [49, 51]); adhesion formation; pelvic pain; voiding problems (b [41, 49]); future fertility problems (b [49]); future pregnancy complications; future surgery complications (b [49]); urinary symptoms; subfertility.

Post-operative pain includes post-operative pain (b [41, 79]); post-operative pain relief (b [47, 49]); analgesia use (b [79]); pain scores and analgesia; pain perception; catheterisation pain / discomfort; time to analgesia; duration of analgesia (b [41]).

Process outcomes include readmission rates (b [75]); reclosure of skin incision; urinary catheter removed; placement of nasogastric tube; nasogastric tube removed; nasogastric tube reinsertion; women's views (b [43, 81]); patient satisfaction (b [41, 45, 47]); caregiver satisfaction (b [41, 79]); quality of life (b [63, 75]); patient reported outcomes (b [52]); unplanned high dependency or intensive care admission (b [42]); blood sugar levels (b [45]); IV fluid administration (b [45]); cosmesis; length of follow up; appearance of scar (b [41]).

Breastfeeding includes breastfeeding (b [49, 81]); duration; maternal self-confidence in breastfeeding (b [80]); time to initiation (b [45, 79]); unsuccessful breastfeeding (b [41, 79]; breastfeeding success (b [45]).

Fetal & childhood wellbeing includes mortality (b [49, 54, 80, 81]); incidence of infant morbidity; delivery time; symptoms of infection; weight; jaundice (b [80, 81]); maximum serum bilirubin levels; phototherapy (b [80]); anaemia (b [51, 81]); haemoglobin (b [81]); haematocrit; polycythaemia; temperature (b [81]); blood transfusion (b [81]); ferritin concentrations (b [81]); intraventricular haemorrhage (b [81]); cost (b [81]); adverse effects (b [44]); oral thrush (b [44]); length of stay (b [44, 49]); immune system development (b [44]); umbilical pH (b [41]); arterial or venous pH; Apgar scores (b [41]); neurodevelopmental outcome (b [43]); periventricular leukomalacia; need for resuscitation (b [43]); hypothermia (b [43]); respiratory distress; use of exogenous surfactant; oxygen dependency; chronic lung disease (b [43]); transfused for low blood pressure; inotropic support for low blood pressure; treatment for patent ductus arteriosus; treatment for hyperbilirubinaemia; blood counts (b [43]); necrotising enterocolitis; birth trauma (b [41, 49]); infant laceration (b [49]); admission to specialist unit (b [49]); readmission (b [49]); hypoxia (b [51]); time to sustained respiration (b [54]); encephalopathy (b [41]); mean regional tissue oxygenation.

Other includes insulin resistance or sensitivity; development of bacterial resistance (b [44]); cost (b [41, 44, 50, 51, 53, 79, 81]); effects on Rhesus-isoimmunisation (b [43]); psychological wellbeing (father) (b [43]); infant bonding (father) (b [43]); anxieties (father) (b [43]); father's views (b [43]); cost-effectiveness (b [63]); ERAS failure (b [63]); length of scar (b [50]); therapeutic uterotonic use.
