# Supplementary material for: Enhanced recovery after elective caesarean: a rapid review of clinical protocols, and an umbrella review of systematic reviews
Source: BMC Pregnancy Childbirth. 2017 Mar 20;17:91. doi: 10.1186/s12884-017-1265-0 (PMC5359888; doi:10.1186/s12884-017-1265-0)
Supplement: Additional file 7: Table S4. — AGREE II criteria for assessing quality of studies of proposed ERAS packages in elective caesarean. Table presenting the results of the quality assessment undertaken in terms of risk of bias of the included studies (ERAS packages in elective caesarean). (PDF 22 kb) [file 12884_2017_1265_MOESM7_ESM.pdf]

Additional file 7 – Table 4: AGREE II criteria for assessing quality of studies of proposed ERAS packages in elective caesarean

AGREE II items	**Lucas** [30]	**Wrench** [32]	**Halder** [31]	**Abell / Long** [34–36]	**Damluji** [33]
**Domain 1. Scope and Purpose**	**43%**	**67%**	**71%**	**62%**	**67%**
1. The overall objective(s) of the guideline is (are) specifically described.	6	7	7	6	6
2. The health question(s) covered by the guideline is (are) specifically described.	1	1	1	1	1
3. The population (patients, public etc) to whom the guideline is meant to apply is specifically described.	2	6	7	6	7
**Domain 2. Stakeholder involvement**	**14%**	**100%**	**62%**	**24%**	**19%**
4. The guideline development group includes individuals from all the relevant professional groups.	1	7	4	2	1
5. The views and preferences of the target population (patients, public etc) have been sought	1	7	2	1	1
6. The target users of the guideline are clearly defined.	1	7	7	2	2
**Domain 3. Rigour of development**	**14%**	**14%**	**23%**	**14%**	**14%**
7. Systematic methods were used to search for evidence.	1	1	1	1	1
8. The criteria for selecting the evidence are clearly used.	1	1	1	1	1
9. The strengths and limitations of the body of evidence are clearly described.	1	1	1	1	1
10. The methods for formulating the recommendations are clearly described.	1	1	1	1	1
11. The health benefits, side effects and risks have been considered in formulating the recommendations.	1	1	6	1	1
12. There is an explicit link between the recommendation and the supporting evidence.	1	1	1	1	1
13. The guideline has been externally reviewed by experts prior to its publication	1	1	1	1	1
14. A procedure for updating the guideline is provided.	1	1	1	1	1
**Domain 4. Clarity of presentation**	**67%**	**57%**	**100%**	**52%**	**38%**
15. The recommendations are specific and unambiguous.	6	4	7	6	6
16. The different options for management of the condition or health issue are clearly presented.	1	1	7	1	1
17. Key recommendations are easily identifiable.	7	7	7	4	1
**Domain 5. Applicability**	**39%**	**29%**	**57%**	**14%**	**14%**
18. The guideline provides advice and/or tools on how the recommendations can be put into practice	1	2	7	1	1
19. The guideline described facilitators and barriers to its application.	7	3	1	1	1
20. The potential resource implications of applying the recommendations have been considered.	2	2	1	1	1

21. The guideline presents monitoring and/or auditing criteria.	1	1	7	1	1
**Domain 6. Editorial Independence**	**57%**	**100%**	**57%**	**14%**	**14%**
22. The views of the funding body have not influenced the context of the guideline.	7	7	7	1	1
23. Competing interests of guideline development group members have been recorded and addressed.	1	7	1	1	1
**TOTAL**	**39%**	**61%**	**62%**	**30%**	**28%**
