# Supplementary material for: Enhanced recovery after elective caesarean: a rapid review of clinical protocols, and an umbrella review of systematic reviews
Source: BMC Pregnancy Childbirth. 2017 Mar 20;17:91. doi: 10.1186/s12884-017-1265-0 (PMC5359888; doi:10.1186/s12884-017-1265-0)
Supplement: Additional file 8: Table 5. — Aggregate Summary of Findings table. Table presenting the summary of findings from the included studies (ERAS components and packages in any setting). (PDF 57 kb) [file 12884_2017_1265_MOESM8_ESM.pdf]

Additional file 8 – Table 5: Aggregate Summary of Findings table

Author of Paper	Component/ Package	Illustrative comparative risks (95% CI)		No of Participants (studies)	Quality of evidence (GRADE)	Comments
		Assumed risk [control]	Corresponding risk [experimental]			
N/A	**Patient selection**					No systematic reviews identified in relation to this component.
N/A	**Perioperative information**					No systematic reviews identified in relation to this component.
Smith [39] (elective surgery)	**Preoperative drinks** (Carbohydrate drink)	Mean hospital LoS ranged across control groups from 1 to 16 days	Mean hospital LoS in the intervention group was 0.30 days lower (0.56 days lower to 0.04 days lower)	n= 1351 (19 studies)<br><br>(27 studies in total but only 19 reported on LoS)	⊕⊖⊖⊖ very low	Substantial heterogeneity Publication bias
Burden [40] (GI surgery)	(Immune enhancing drink)	Mean hospital LoS in the control groups was 15.3 days (ranged from 9.8 to 25 days)	Mean hospital LoS in the intervention groups was 0.97 days lower (1.64 days lower to 0.30 days lower)	n=549 (6 studies)<br><br>(13 studies in total but only 6 reported on LoS)	⊕⊕⊕⊖ moderate	Low to moderate heterogeneity No blinding Publication bias
N/A	**Preoperative fluids**					No systematic reviews identified in relation to this component.
N/A	**Perioperative fluid balance**					No systematic reviews identified in relation to this component.
Mathai [41] (caesarean section)	**Surgical technique** (Joel Cohen vs. Pfannensteil incision)	Mean hospital LoS in Pfannensteil group was 5.9 days	Mean hospital LoS in the Joel Cohen group was 1.5 days lower (2.16 days lower to 0.84 days lower)	n=101 (1 study)<br><br>(2 studies in total focusing on this technique, but only 1 reported on LoS)	⊕⊖⊖⊖ very low	Small sample size Single study No blinding

	(Muscle-cutting vs. Pfannensteil incision)	Mean hospital LoS in Pfannensteil group was 6.3 days	Mean hospital LoS in the muscle-cutting group was 0.4 days higher (0.34 lower to 1.14 days higher)	n=97 (1 study)<br><br>(2 studies in total focusing on this technique, but only 1 reported on LoS)	⊕⊖⊖⊖ very low	Small sample size<br>Single study<br>No blinding
Dodd [49] (caesarean section)	(Single layer uterine closure vs. double layer)	Mean hospital LoS in double layer group was 4.9 days	Mean hospital LoS in single layer group was 0.10 days lower (0.52 days lower to 0.32 days higher)	n=158 (1 study)<br><br>(19 studies in total focusing on this technique, but only 1 reported on LoS)	⊕⊖⊖⊖ very low	Small sample size<br>Single study<br>No blinding
Mackeen [50] (caesarean section)	(Staples vs. subcuticular stitch)	Mean hospital LoS in subcuticular group was 3.1 days	Mean hospital LoS in staples group was 0.10 days higher (0.01 days lower to 0.21 days higher)	n=416 (1 study)<br><br>(6 studies in total focusing on this technique, but only 1 reported on LoS)	⊕⊖⊖⊖ very low	Single study<br>Unclear blinding<br>Incomplete data
Jacobs-Jokhan [79] (caesarean section)	(Extra-abdominal vs. intra-abdominal repair of the uterine incision)	Mean postoperative LoS ranged across intra-abdominal group from 3.45 to 5.6 days	Mean postoperative LoS in the extra-abdominal group was 0.24 days higher (0.08 days higher to 0.39 days higher)	n=766 (4 studies)<br><br>(6 studies in total but only 4 reported on LoS)	⊕⊕⊕⊖ moderate	All four studies were RCTs<br>Uncertainty over allocation concealment in most studies
Alderson [42] (elective surgery)	**Patient warming**	Mean post anaesthesia care unit (PACU) LoS in control group was 160 minutes	Mean hospital post anaesthesia care unit (PACU) LoS in the intervention group was 9 minutes lower (45.32 minutes lower to 27.32 minutes higher)	n=40 (1 study)<br><br>(22 studies in total but only 1 reported on LoS)	⊕⊖⊖⊖ very low	Small sample size<br>Single study<br>No blinding<br>Wide CI with data from small number of patients in study<br>Unclear allocation concealment
N/A	**Perioperative analgesia**					No systematic reviews identified in relation to this component.

Rabe [43] (caesarean section / vaginal birth)	**Delayed umbilical cord clamping**	Mean hospital LoS (infant only) in control group was 56.6 days	Mean hospital LoS (infant only) in the intervention group was 16.40 days lower (38.06 days lower to 5.26 days higher)	n=32 (1 study)<br><br>(15 studies in total but only 1 reported on LoS)	⊕⊕⊖⊖ low	Low population Single study
N/A	**Prevention of post operative nausea**					No systematic reviews identified in relation to this component.
Smaill [44] (caesarean section)	**Postoperative antibiotic prophylaxis**	Mean hospital LoS ranged across control group from 5.24 to 12.1 days	Mean hospital LoS in the intervention group was 0.49 days lower (0.68 days lower to 0.29 days lower)	n=3119 (17 studies)<br><br>(86 studies in total but only 17 reported on LoS)	⊕⊕⊕⊖ moderate	Low to moderate heterogeneity Potential for publication bias
Mangesi [45] (caesarean section)	**Early postoperative fluids/food**	Mean postoperative LoS ranged across control groups from 3.1 to 3.7 days	Mean postoperative LoS in the intervention group was 0.46 days lower (1.21 days lower to 0.30 days higher)	n=338 (3 studies)<br><br>(6 studies in total but only 3 reported on LoS)	⊕⊕⊖⊖ low	Substantial heterogeneity Small number of studes
Andersen [46] (colorectal surgery)		Mean hospital LoS ranged across control group from 6.8 to 16 days	Mean hospital LoS in the intervention group was 0.89 days lower (1.58 days lower to 0.20 days lower)	n=1132 (14 studies)	⊕⊕⊕⊖ moderate	Evidence of heterogeneity
Charoenkwan [55] (gynecologic surgery)		Mean hospital LoS ranged across control group from 3.64 to 5.8 days (fixed effect model)<br><br>Mean hospital LoS ranged across control group from 3.64 to 5.8 days (random effect model)	Mean hospital LoS in the intervention group was 0.50 days lower (0.75 days lower to 0.24 days lower) (fixed effect model)<br><br>Mean hospital LoS in the intervention group was 0.74 days lower (1.52 days lower to 0.07 days lower) (random effect model)	n=301 (2 studies)<br><br>(3 studies in total but only 2 reported on LoS)	⊕⊕⊕⊖ moderate	Small sample size

Ho [56] (colorectal surgery)	**Sham feeding**	Unknown	Mean hospital LoS in the intervention group was 0.5 days lower (0.86 days lower to 0.14 days lower)	n=535 (8 studies)<br><br>(10 studies in total but only 8 reported on LoS)	⊕⊕⊖⊖ low	Substantial heterogeneity<br>Some small sample sizes<br>All eight studies were RCTs
Abdel-Aleem [47] (caesarean section)	**Early removal of catheter** (Indwelling catheter vs. no catheter)	Mean hospital LoS ranged across control group from 1.88 to 3.06 days	Mean hospital LoS in the intervention group was 0.62 days higher (0.15 days higher to 1.10 days higher)	n=840 (3 studies)<br><br>(5 studies in total but only 3 reported on LoS)	⊕⊕⊖⊖ low	Substantial heterogeneity<br>Small number of studies
N/A	**Postoperative mobilization**					No systematic reviews identified in relation to this component.
Nicholson [57] (elective surgery)	**Enhanced recovery after surgery (ERAS) package**	Median LoS ranged across control groups from 3 to 11 days	Mean hospital LoS in the intervention groups was 1.14 days lower (1.45 days lower to 0.85 days lower)	n=2995 (23 studies)<br><br>(38 studies in total but only 23 reported on LoS)	⊕⊕⊖⊖ low	Substantial heterogeneity<br>No blinding
Spanjersberg [63] (colorectal surgery)		Mean hospital LoS ranged across control groups from 7 to 10.4 days	Mean hospital LoS in the intervention groups was 2.94 days lower (3.69 days lower to 2.19 days lower)	n=237 (4 studies)<br><br>(6 studies in total but only 4 reported on LoS)	⊕⊖⊖⊖ very low	Substantial heterogeneity<br>Low sample size<br>No blinding<br>Lack of clarity between research question and primary outcome
Markar [59] (esophagectomy)		Unknown	Mean hospital LoS in the intervention groups was 2.28 days lower (3.12 days lower to 1.43 days lower)	n=1240 (9 studies)	⊕⊕⊕⊖ moderate	Substantial heterogeneity<br>High risk of bias
Zhuang [62] (colorectal surgery)		Mean primary hospital LoS ranged across control groups from 4 to 7.4 days<br><br>(Total hospital LoS also reported but not included here)	Mean primary hospital LoS in the intervention groups was 2.44 days lower (3.06 days lower to 1.83 days lower)<br><br>(Total hospital LoS also reported but not included here)	n=1740 (11 studies)<br><br>(13 studies in total but only 11 reported on primary hospital LoS)	⊕⊕⊖⊖ low	Some heterogeneity<br>High risk of bias<br>No blinding<br>Publication bias

Varadhan [12] (colorectal surgery)		Mean hospital LoS ranged across control groups from 5.8 to 10.4 days	Mean hospital LoS in the intervention groups was 2.51 days lower (3.54 days lower to 1.47 days lower)	n=452 (6 studies)	⊕⊕⊖⊖ low	Substantial heterogeneity<br>Lack of study data<br>Significant risk of bias<br>All six studies were RCTs
Walter [72] (colorectal resections)		Mean total hospital LoS ranged across control groups from 8 to 21 days	Mean total hospital LoS in the intervention groups was 3.75 days lower (5.11 days lower to 2.40 days lower)	n=376 (4 studies)	⊕⊕⊕⊖ moderate	Low to moderate heterogeneity<br>Two studies were RCTs
Lv [73] (colorectal surgery)		Mean total hospital LoS ranged across control groups from 6 to 10.4 days	Mean total hospital LoS in the intervention groups was 1.88 days lower (2.91 days lower to 0.86 days lower)	n=852 (7 studies)	⊕⊕⊖⊖ low	Moderate risk of bias<br>No blinding<br>All seven studies were RCTs<br>Substantial heterogeneity
Adamina [66] (colorectal surgery)		Median hospital LoS ranged across control groups (where reported) from 7 to 9 days	Median hospital LoS in the intervention groups was 2.5 days lower (3.92 days lower to 1.11 days lower)	n=452 (6 studies)	⊕⊕⊕⊖ moderate	All studies were RCTs<br>Wide CI with mainly small sample sizes
Wind [65] (colonic surgery)		Mean primary hospital LoS ranged across control groups from 5.8 to 10 days	Mean primary hospital LoS in the intervention groups was 1.56 days lower (2.61 days lower to 0.50 days lower)	n=460 (5 studies)<br><br>(6 studies in total but only 5 reported on LoS)	⊕⊕⊖⊖ low	Several sources of bias, potential for publication bias<br>Substantial heterogeneity<br>Three studies were RCTs
Gouvas [74] (colorectal surgery)		Mean total hospital LoS ranged across control groups (where reported) from 6.5 to 13 days	Mean total hospital LoS in the intervention groups was 2.46 days lower (3.43 days lower to 1.48 days lower)	n=639 (5 studies)<br><br>(11 studies in total but only 5 reported on LoS)	⊕⊕⊖⊖ low	Two studies were RCTs<br>Some small sample sizes<br>No blinding

Please note that 15 studies were not presented in the above table as they did not report sufficiently, on length of stay. These were: Hughes [60]**, Kagedan** [58]**, Bamigboye** [53]**, Munday** [37, 38]**, Hall** [69]**, Lemmens** [70]**, Eskicioglu** [64]**, Ahmed** [67]**, Coolsen** [61]**, Lv** [75]**, Sturm** [76]**, Coolsen** [68]**, Rawlinson** [71]**, Billson** [48] **and Becker** [80]**. A further 6 studies were not presented as they did not report on length of stay: McDonald** [51]**, Urrútia** [52]**, Afolabi** [54]**, Khan** [77]**, Palethorpe** [81]**, and Sia** [78]**.**
