# Supplementary material for: Enhanced recovery after elective caesarean: a rapid review of clinical protocols, and an umbrella review of systematic reviews
Source: BMC Pregnancy Childbirth. 2017 Mar 20;17:91. doi: 10.1186/s12884-017-1265-0 (PMC5359888; doi:10.1186/s12884-017-1265-0)
Supplement: Additional file 9: Table 6. — AMSTAR Assessment. Table presenting the results of the quality assessment undertaken in terms of risk of bias of the included studies (ERAS components and packages in any setting). (PDF 45 kb) [file 12884_2017_1265_MOESM9_ESM.pdf]

Additional file 9 – Table 6: AMSTAR Assessment

AMSTAR Items	**Smith** [39] *(elective surgery)*	**Burden** [40] *(GI surgery)*	**Mathai** [41] *(caesarean section)*	**Alderson** [42] *(elective surgery)*	**Rabe** [43] *(caesarean section / vaginal birth)*	**Smaill** [44] *(caesarean section)*	**Mangesi** [45] *(caesarean section)*	**Andersen** [46] *(colorectal surgery)*	**Abdel-Aleem** [47] *(caesarean section)*	**Billson** [48] *(ovarian cancer)*
1. Was an 'a priori' design provided?	Yes	Yes	Yes	Yes	Yes	Yes	Yes	Yes	Yes	Yes
2. Was there duplicate study selection and data extraction?	Yes	Yes	Yes	Yes	Yes	Yes	Can't answer	Yes	Yes	Yes
3. Was a comprehensive literature search performed?	Yes	Yes	Yes	Yes	Yes	Yes	Yes	Yes	Yes	Yes
4. Was the status of publication (i.e. grey literature) used as an inclusion criterion?	Yes	Can't answer	Yes	Yes	Yes	Yes	Yes	Yes	Yes	Yes
5. Was a list of studies (included and excluded) provided?	Yes	Yes	Yes	Yes	Yes	Yes	Yes	Yes	Yes	Yes
6. Were the characteristics of the included studies provided?	Yes	Yes	Yes	Yes	Yes	Yes	Yes	Yes	Yes	Yes
7. Was the scientific quality of the included studies assessed and documented?	Yes	Yes	Yes	Yes	Yes	Yes	Can't answer	Yes	Yes	Yes
8. Was the scientific quality of the included studies used appropriately in formulating conclusions?	Yes	Yes	Can't answer	Yes	Yes	Yes	Yes	Yes	Yes	Yes
9. Were the methods used to combine the findings of studies appropriate?	Yes	Yes	N/A	N/A	N/A	Yes	Yes	Yes	N/A	N/A
10. Was the likelihood of publication bias assessed?	Yes	No	No	No	Yes	Yes	No	Yes	No	No
11. Was the conflict of interest included?	Yes	Yes	Yes	Yes	Yes	No	No	No	No	Yes

AMSTAR Items	**Dodd** [49] *(caesarean section)*	**Mackeen** [50] *(caesarean section)*	**McDonald** [51] *(caesarean section / vaginal birth)*	**Urrútia** [52] *(elective surgery)*	**Bamigboye** [53] *(caesarean section)*	**Afolabi** [54] *(caesarean section)*	**Charoenkwan** [55] *(gynaecologic surgery)*	**Munday** [37, 38] *(caesarean section)*	**Ho** [56] *(colorectal surgery)*	**Nicholson** [57] *(elective surgery)*
1. Was an 'a priori' design provided?	Yes	Yes	Yes	Yes	Yes	Yes	Yes	Yes	Yes	Yes
2. Was there duplicate study selection and data extraction?	Yes	Yes	Yes	Yes	Yes	Yes	Yes	Can’t answer	Can’t answer	Yes
3. Was a comprehensive literature search performed?	Yes	Yes	Yes	Yes	Yes	Yes	Yes	Yes	No	Yes
4. Was the status of publication (i.e. grey literature) used as an inclusion criterion?	Yes	Yes	Yes	Yes	Yes	Yes	Can’t answer	Yes	No	Can’t answer
5. Was a list of studies (included and excluded) provided?	Yes	Yes	Yes	Yes	Yes	Yes	Yes	Yes	No (only included studies)	Can’t answer
6. Were the characteristics of the included studies provided?	Yes	Yes	Yes	Yes	Yes	Yes	Yes	Yes	Yes	No
7. Was the scientific quality of the included studies assessed and documented?	Yes	Yes	Yes	Yes	Yes	Yes	Yes	Yes	Yes	No
8. Was the scientific quality of the included studies used appropriately in formulating conclusions?	Yes	Yes	Yes	Yes	Yes	Yes	Yes	Yes	Yes	No
9. Were the methods used to combine the findings of studies appropriate?	N/A	N/A	N/A	N/A	N/A	N/A	Yes	Yes	No	Yes
10. Was the likelihood of publication bias assessed?	Yes	Yes	Yes	Yes	Yes	No	Yes	No	Yes	Yes
11. Was the conflict of interest included?	Yes	Yes	Yes	Yes	Yes	Yes	Yes	No	No	Yes

AMSTAR Items	**Kadegan** [58] *(pancreatic surgery)*	**Markar** [59] *(esophagectomy)*	**Hughes** [60] *(liver surgery)*	**Coolsen** [61] *(pancreatic surgery)*	**Zhuang** [62] *(colorectal surgery)*	**Spanjersberg** [63] *(colorectal surgery)*	**Eskicioglu** [64] *(colorectal surgery)*	**Wind** [65] *(colonic surgery)*	**Adamina** [66] *(colorectal surgery)*	**Ahmed** [67] *(colorectal surgery)*
1. Was an 'a priori' design provided?	Can't answer	Can't answer	Can't answer	Can't answer	Can't answer	Yes	Can't answer	Can't answer	Can't answer	Can't answer
2. Was there duplicate study selection and data extraction?	Can't answer	Yes	Yes	Yes	Can't answer	Can't answer	Yes	Can't answer	Yes	Can't answer
3. Was a comprehensive literature search performed?	Yes	Yes	Yes	Yes	Yes	Yes	Yes	Yes	Yes	Yes
4. Was the status of publication (i.e. grey literature) used as an inclusion criterion?	Can't answer	Yes	Can't answer	Can't answer	Can't answer	Yes	Yes	No	No	No
5. Was a list of studies (included and excluded) provided?	No (only included studies)	No	No (only included studies)	No	No	Yes	Yes	Yes	Yes	Yes
6. Were the characteristics of the included studies provided?	Yes	No	Yes	Yes	Yes	Yes	Yes (participant numbers unclear)	Yes	No	Yes
7. Was the scientific quality of the included studies assessed and documented?	Yes	Yes	No	Yes	Yes	Yes	No (assessed but not documented)	No (assessed but not documented)	No (assessed but not documented)	No
8. Was the scientific quality of the included studies used appropriately in formulating conclusions?	Yes	Yes	No	Yes	Yes	Yes	No	No	No	No
9. Were the methods used to combine the findings of studies appropriate?	Yes	Yes	N/A	Can't answer	Can't answer	Yes	Yes	Yes	Yes	Can't answer
10. Was the likelihood of publication bias assessed?	No	Yes	No	Yes	Yes	Yes	No (funnel plots mentioned but not documented)	No	No	No
11. Was the conflict of interest included?	Yes	Yes	Yes	Yes	Yes	Yes	No	No	No	No

AMSTAR Items	**Coolsen** [68] *(liver surgery)*	**Hall** [69] *(hepatobiliary & pancreatic surgery)*	**Lemmens** [70] *(digestive surgery)*	**Rawlinson** [71] *(colorectal surgery)*	**Varadhan** [12] *(colorectal surgery)*	**Walter** [72] *(colorectal resections)*	**Lv** [73] *(colorectal surgery)*	**Gouvas** [74] *(colorectal surgery)*	**Lv** [75] *(gynaecological cancer)*	**Sturm** [76] *(fast-track surgery)*
1. Was an 'a priori' design provided?	Can’t answer	Can’t answer	Can’t answer	Can’t answer	Can’t answer	Can’t answer	Can’t answer	Can’t answer	Can’t answer	Can’t answer
2. Was there duplicate study selection and data extraction?	Can’t answer	Can’t answer	Yes	Can’t answer	Yes	Can’t answer	Yes	Can’t answer	Yes	No
3. Was a comprehensive literature search performed?	Yes	No	No	No	Yes	Yes	Yes	Yes	Yes	Yes
4. Was the status of publication (i.e. grey literature) used as an inclusion criterion?	No	No	No	No	Yes	No	No	No	Yes	Yes
5. Was a list of studies (included and excluded) provided?	Yes	Yes	No	Yes	No	No	No	Yes	N/A	No
6. Were the characteristics of the included studies provided?	Yes	Yes	Yes	Yes	Yes	Yes	Yes	Yes	N/A	Yes
7. Was the scientific quality of the included studies assessed and documented?	No (assessed but not documented)	No	No	No	Yes	Yes	Yes	Yes	Yes	Yes
8. Was the scientific quality of the included studies used appropriately in formulating conclusions?	No	No	No	No	Yes	Yes	No	No	N/A	Yes
9. Were the methods used to combine the findings of studies appropriate?	Can’t answer	Can’t answer	Can’t answer	Can’t answer	Yes	Yes	Yes	Yes	N/A	Can’t answer
10. Was the likelihood of publication bias assessed?	No	No	No	No	No	Yes	Yes	No (funnel plots mentioned but not documented)	Yes	No
11. Was the conflict of interest included?	No	No	No	No	No	No	No	No	Yes	No

**AMSTAR Items**	**Khan** [77] *(colorectal surgery & enhanced recovery)*	**Palethorpe** [81] *(caesarean section / vaginal birth)*	**Sia** [78] *(caesarean section)*	**Jacobs-Jokhan** [79] *(caesarean section)*	**Becker** [80] *(caesarean section)*
1. Was an 'a priori' design provided?	Can't answer	Yes	Yes	Yes	Yes
2. Was there duplicate study selection and data extraction?	Can't answer	Can't answer	Yes	Can't answer	Yes
3. Was a comprehensive literature search performed?	Yes	No	Yes	Yes	Yes
4. Was the status of publication (i.e. grey literature) used as an inclusion criterion?	No	Yes	Yes	Yes	Yes
5. Was a list of studies (included and excluded) provided?	No	Yes	Yes	Yes	Yes
6. Were the characteristics of the included studies provided?	Yes	N/A	Yes	Yes	Yes
7. Was the scientific quality of the included studies assessed and documented?	Yes	N/A	Yes	Yes	Yes
8. Was the scientific quality of the included studies used appropriately in formulating conclusions?	Yes	N/A	Yes	Yes	Yes
9. Were the methods used to combine the findings of studies appropriate?	Yes	N/A	Yes	Yes	Yes
10. Was the likelihood of publication bias assessed?	No	N/A	No	No	Yes
11. Was the conflict of interest included?	No	Yes	No	No	No
