# Supplementary figures and images for: Enhanced recovery after elective caesarean: a rapid review of clinical protocols, and an umbrella review of systematic reviews
Source: BMC Pregnancy Childbirth. 2017 Mar 20;17:91. doi: 10.1186/s12884-017-1265-0 (PMC5359888; doi:10.1186/s12884-017-1265-0)

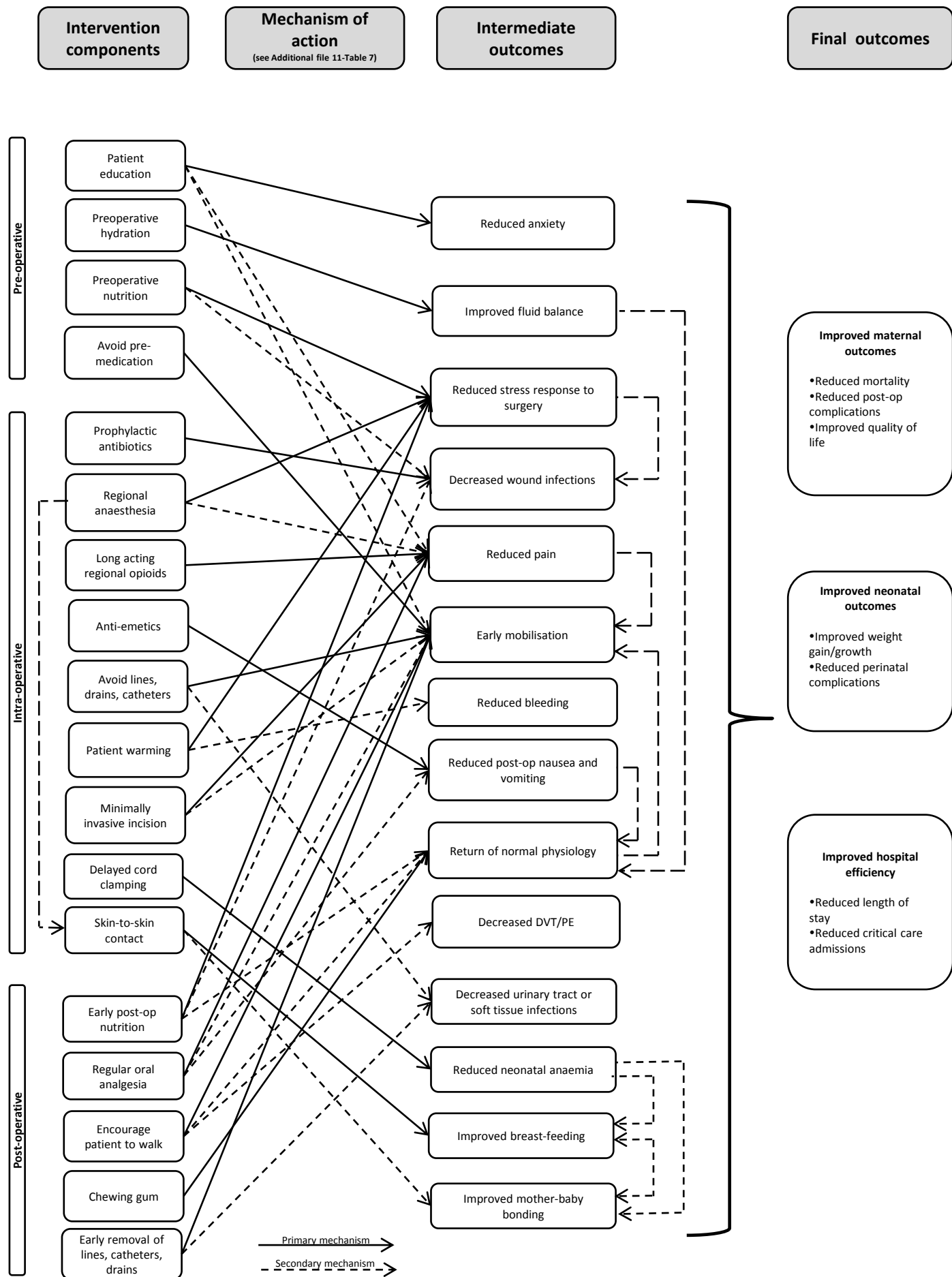

Supplement: Additional file 10: Figure S3. — Proposed mechanisms of action for broad categories of ERAS components. Figure illustrating how the proposed mechanisms of action may work in an enhanced recovery pathway. (PDF 212 kb) [file 12884_2017_1265_MOESM10_ESM.pdf]
