# Supplementary material for: Enhanced recovery after elective caesarean: a rapid review of clinical protocols, and an umbrella review of systematic reviews
Source: BMC Pregnancy Childbirth. 2017 Mar 20;17:91. doi: 10.1186/s12884-017-1265-0 (PMC5359888; doi:10.1186/s12884-017-1265-0)
Supplement: Additional file 11: Table S7. — Interventions, mechanisms and actions. Table detailing the theory of the proposed mechanisms of action in an enhanced recovery pathway. (PDF 22 kb) [file 12884_2017_1265_MOESM11_ESM.pdf]

Additional file 11 – Table 7: Interventions, mechanisms and actions

Phase of Operation	Intervention	Mechanism of action	Intermediate actions
Preoperative	Patient education	Increases patient knowledge about procedure and what to expect	Reduced anxiety, improved pain control, aware of milestones to discharge → **Reduced LoS**
	Hydration (clear oral fluids up to two hours preoperatively)	Reduces the chances of postoperative dehydration.	Less need for postoperative intravenous fluids with earlier discontinuation of them →**Reduced LoS**
	Nutrition and carbohydrate drinks	Reduces the insulin resistance associated with surgery.	Fewer postoperative infections and less breakdown of muscle protein. There are also reduced feelings of hunger and thirst and patients are less anxious → **Reduced LoS**
	Avoidance of premedication	Avoids long acting sedatives such as diazepam	Reduces psychomotor impairment during the postoperative period. Early mobilization → **Reduced LoS**
	Prophylactic antibiotics	Prevents infection at site of surgical incision	Reduces postoperative complications → **Reduced LoS**
Intra operative	Spinal (regional) anaesthesia	Reduces the body's stress response compared to general anaesthetic	Patient experiences less stress and so recovers more quickly
	Long acting regional opioids (opioids added to local anaesthetic for spinal anaesthesia)	Reduces postoperative pain and consequently reduces the demand for morphine injections	Fewer side effects from strong morphine like pain killers (sleepiness, nausea etc) enabling earlier mobilization → **Reduced LoS**
	Anti-sickness drugs	Reduces postoperative nausea and vomiting	Reduced nausea, increased mobilization → **Reduced LoS**
	Patient warming	Prevents postoperative hypothermia	Reduced stress response and fewer wound infections, bleeding and heart problems → **Reduced LoS**
	Minimally invasive surgical technique: Horizontal lower abdominal incision for surgery (national standard)	Reduces postoperative pain thus reduces the need for morphine injections to control it	There is less need for strong pain killers that would have side effects, keeping patients in bed for longer. Early mobilization → **Reduced LoS**

	Avoidance of lines, drains and urinary catheters.		
	Delayed clamping of the umbilical cord on delivery of the baby	Increases blood flow from the placenta into the baby.	Reduced incidence of anaemia in baby.
	Skin to skin contact at birth	Improves mother and baby bonding	Early start of breastfeeding → **Reduced LoS**
Postoperative	Analgesia<br>controlled with oral medication – regular paracetamol and brufen with oral morphine for breakthrough pain	Reduces the need for morphine injections to control pain	Fewer side effects from morphine injections which might prevent the mother from getting out of bed.<br>Early mobilisation → **Reduced LoS**
	Early removal of urinary catheters.	Reduces the chance of developing a urinary tract infection.	Reduced rate of urinary tract infection. Also allows for earlier mobilization → **Reduced LoS**
	Early oral intake	Maintains the mother's blood glucose levels so that muscle is not broken down to provide energy.	Fewer postoperative infections and less breakdown of muscle protein. There are also reduced feelings of hunger and thirst and patients are less anxious.<br>Early return of gut function.→ **Reduced LoS**
	Early mobilization	Prevents muscle wasting and encourages the flow of blood in the legs.	Fewer blood clots in legs and lungs and improved lung and gut function.<br>Early return to normal function. → **Reduced LoS**
	Chewing gum	Stimulates bowel movements after abdominal surgery.	Early return of bowel function. Early return of normal function → **Reduced LoS**
