# Supplementary material for: Enhanced recovery after elective caesarean: a rapid review of clinical protocols, and an umbrella review of systematic reviews
Source: BMC Pregnancy Childbirth. 2017 Mar 20;17:91. doi: 10.1186/s12884-017-1265-0 (PMC5359888; doi:10.1186/s12884-017-1265-0)
Supplement: Additional file 12: Table S8. — Proposed barriers and facilitators of uptake. Table detailing the barriers and facilitators of adoption of healthcare innovations. (PDF 19 kb) [file 12884_2017_1265_MOESM12_ESM.pdf]

Additional file 12 – Table 8: Proposed barriers and facilitators of uptake

**Targets of the KT intervention** [94]	**Matching facilitators of change, identified in ER pathway implementation studies** [17]
1. Factors in health professionals	
Learn about the new knowledge	Multidisciplinary training days [113]; teaching sessions with all new nurses and doctors [114]
Influence the views of others regarding the new knowledge	All stakeholders involved from the planning stage [113]; ensure commitment to change from all stakeholders [99]
2. Factors in patient care teams and networks	
Change structures and process in teams	All stakeholders involved from the planning stage [113]; use of ERAS link nurses [113]; Combined approach of a clinician and a manager working together to implement the pathways was crucial, due to the large volumes of patients to be treated and the complex organisational issues associated with introducing change and new ways of working [99]; a highly organised logistical framework [99]
Use leadership and key individuals	Senior management support [113]; use of ERAS link nurses [113, 114]; management support (see above) [99]; role of specialist bariatric nurses and pharmacists [115]
3. Factors in healthcare organizations	
Change specification (prescription of specific practices) and flexibility (room for deviation from prescribed practices)	Wainwright study argues for the importance of standardised procedures [99], but in other studies, concern was expressed over rigidity of protocolisation. For instance, in the McGeehan report, concern was expressed by anaesthetic staff over patients being in pain, but issues being addressed [100]
Influence leadership in an organisation	Senior management support [113]; importance of co-operation of clinical and management staff (see above).
Implement continuous improvement activities	Continued audit and evaluation [113]
Improve communication within an organisation	Communications programme [113]; regular feedback [114]

References

113. Payne J, Beazley S, Morris H. 4 Winning Principles: Cancer Inpatients Case Series: Enhanced Recovery After Surgery Program – Improving Patient Outcomes for Colorectal Surgical Patients. London: NHS Improvement. 2008. http://webarchive.nationalarchives.gov.uk/20130107105354/http://www.dh.gov.uk/prod_consum_dh/groups/dh_digitalassets/documents/digitalasset/dh_117532.pdf. Accessed November 19, 2015.

114. Elwood M. Implementing an enhanced recovery programme in colorectal surgery. Nursing Times. 2008. http://www.nursingtimes.net/implementing-an-enhanced-recovery-programme-in-colorectal-surgery/1247153.article. Accessed June 23, 2015.

115. Zamoyski T, Khera G, Simpson A, Wilson M, Woodcock S, Seymour K. Enhanced Recovery Program Within a Bariatric Service. North Shields: Department of General Surgery, North Tyneside General Hospital; 2011.
